# Prognosis of the individual course of disease - steps in developing a decision support tool for Multiple Sclerosis

**DOI:** 10.1186/1472-6947-7-11

**Published:** 2007-05-08

**Authors:** M Daumer, A Neuhaus, C Lederer, M Scholz, JS Wolinsky, M Heiderhoff, the Sylvia Lawry Centre for Multiple Sclerosis Research

**Affiliations:** 1 Sylvia Lawry Centre for Multiple Sclerosis Research, Munich, Germany; 2 Trium Analysis Online GmbH, Munich, Germany; 3 University of Texas Health Science Center, Houston, TX, USA; 4 University Hospital Heidelberg, Department of General Practice and Health Services Research, Heidelberg, Germany

## Abstract

**Background:**

Multiple sclerosis is a chronic disease of uncertain aetiology. Variations in its disease course make it difficult to impossible to accurately determine the prognosis of individual patients. The Sylvia Lawry Centre for Multiple Sclerosis Research (SLCMSR) developed an "online analytical processing (OLAP)" tool that takes advantage of extant clinical trials data and allows one to model the near term future course of this chronic disease for an individual patient.

**Results:**

For a given patient the most similar patients of the SLCMSR database are intelligently selected by a model-based matching algorithm integrated into an OLAP-tool to enable real time, web-based statistical analyses. The underlying database (last update April 2005) contains 1,059 patients derived from 30 placebo arms of controlled clinical trials. Demographic information on the entire database and the portion selected for comparison are displayed. The result of the statistical comparison is provided as a display of the course of Expanded Disability Status Scale (EDSS) for individuals in the database with regions of probable progression over time, along with their mean relapse rate. Kaplan-Meier curves for time to sustained progression in the EDSS and time to requirement of constant assistance to walk (EDSS 6) are also displayed. The software-application OLAP anticipates the input MS patient's course on the basis of baseline values and the known course of disease for similar patients who have been followed in clinical trials.

**Conclusion:**

This simulation could be useful for physicians, researchers and other professionals who counsel patients on therapeutic options. The application can be modified for studying the natural history of other chronic diseases, if and when similar datasets on which the OLAP operates exist.

## Background

Multiple sclerosis (MS) is an inflammatory disease of the central nervous system. Currently there is no cure for MS, but there are treatments for its relapsing forms that reduce the short and mid-range impact of the disease. It remains impossible to accurately predict the prognosis of individual patients, which could be important for patient counselling and in weighing therapeutic options. The Sylvia Lawry Centre for MS Research in Munich (SLCMSR) uses data from clinical trials and natural history studies to develop mathematical models for the course of the disease which might assist in the efforts to accurately determine short, mid and long-range prognosis. The "individual risk profile" project makes the Centre's large database available to health care professionals via access to a representative part of the database.

## Implementation

The SLCMSR database consist of data from most natural history studies and the placebo arms of nearly all randomised controlled clinical trials in MS patients from the last decade. The data are anonymised, homogenized and pooled from the academic and corporate sources. As of April 2005, 45 separate data sets and 81,000 patient-years of data from 20,000 patients were included. The database is divided into so-called "open" and "closed" parts. The "open" database is accessible by researchers (and in the future possibly other professionals) at their discretion. The "closed" database is accessible only to authorized staff on behalf of the Validation Committee of the Centre as part of the validation policy of the SLCMSR [1].

An interdisciplinary team of neurologists, information technology specialists, and biostatisticians developed a Java-based OLAP tool using the statistical software package R 2.0.1 [2,3]. The source code is available in Additional file 1. For validation purposes essential parts were independently re-programmed using SAS/INTRNET [4]. Remote users are able to trigger powerful statistical software programs running on the host computer using a simple graphical interface displayed in a standard browser. The results of the analysis are displayed in real time.

Registered OLAP users access the database via their internet browser, select values for patient characteristics of their interest and get displays of the observed disease course for placebo patients from the database with matching characteristics.

## Results

The essential component of the OLAP is a matching algorithm. A given patient of interest is "identified" by a set of covariates which are widely accepted to be potential prognostic factors [5-7]: the number of relapses in the last 12 months, disease duration from diagnosis, age at disease onset, disability level as measured by the Expanded Disability Status Scale (EDSS) [8], and disease course [9] – either relapsing remitting, secondary progressive, primary progressive or clinically isolated syndrome [10,11]. The matching algorithm then automatically selects the most similar subgroup of patients that are included in the banked database, with similarity defined by the covariates on a disease course specific outcome. The resulting display shows the disease course of all patients in the database that are similar to the patient of interest, and this information can be used to project a hypothesized outcome for the patient of interest, based on the behaviour of similar patients in the database.

This descriptive report is based on 1,059 patients from placebo arms of controlled clinical trials available from release 1.0 of the SLCMSR database (April 2005).

**Figure 1:**
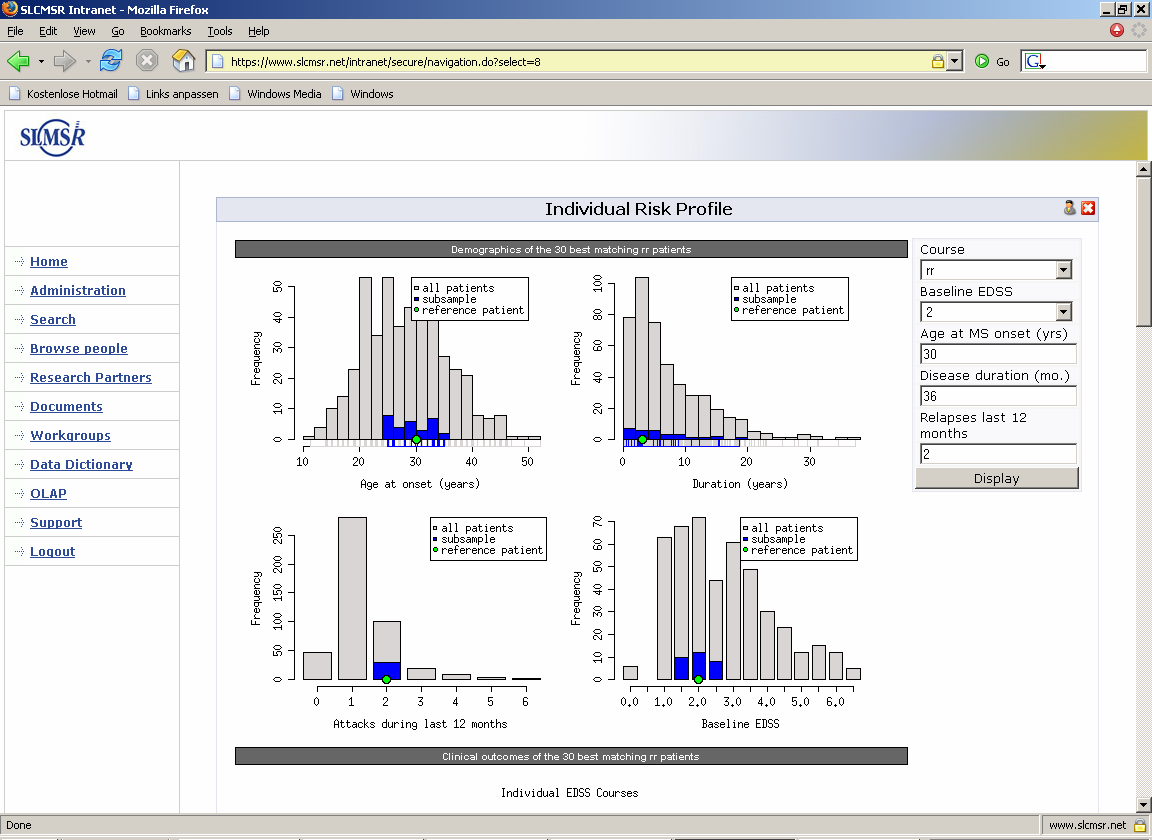
Screenshot from the OLAP-tool "Individual Risk Profile" displaying the demographics of the selected subgroup (blue). The green dot indicates the requested patient characteristics (RR disease course, baseline EDSS 2, age at onset 30 years, disease duration 36 months and 2 relapses in the last 12 months). In addition the distributions of all patients from the clinical trial data having the same course of the disease are displayed to give the user the impression on how representative the selected subgroup for the selected course is (gray).

**Figure 2:**
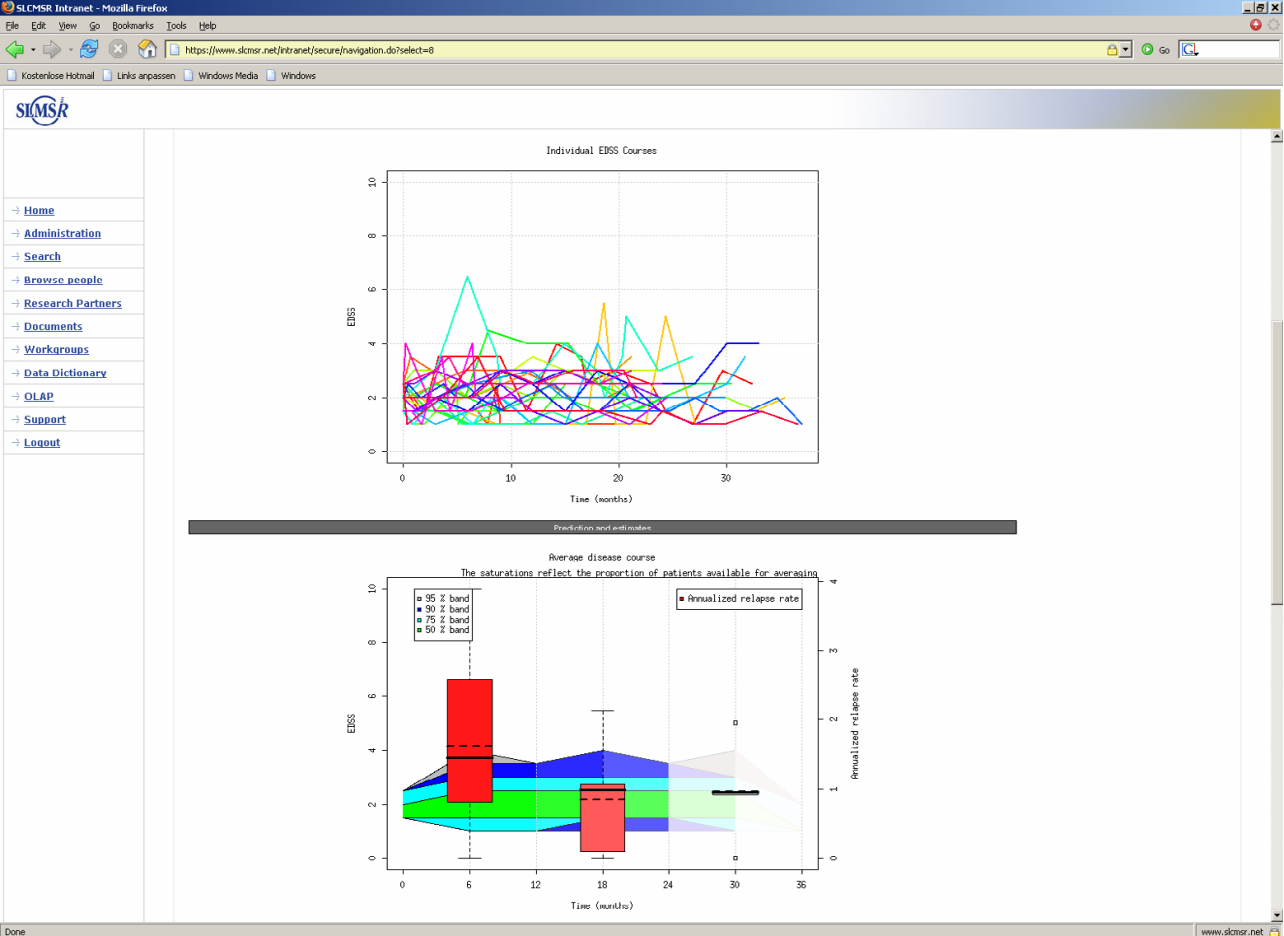
Screenshot from the OLAP-tool "Individual Risk Profile" displaying EDSS courses and regions of EDSS evolution. The interior band defines the middle 50% of EDSS courses. The other bands outline (from the inside to the outside) 75%, 90% and 95% of the individual disease courses. Additional information about the disease course is provided by the annual relapse rates. These are displayed as box plots and illustrate the disease activity.

**Figure 3:**
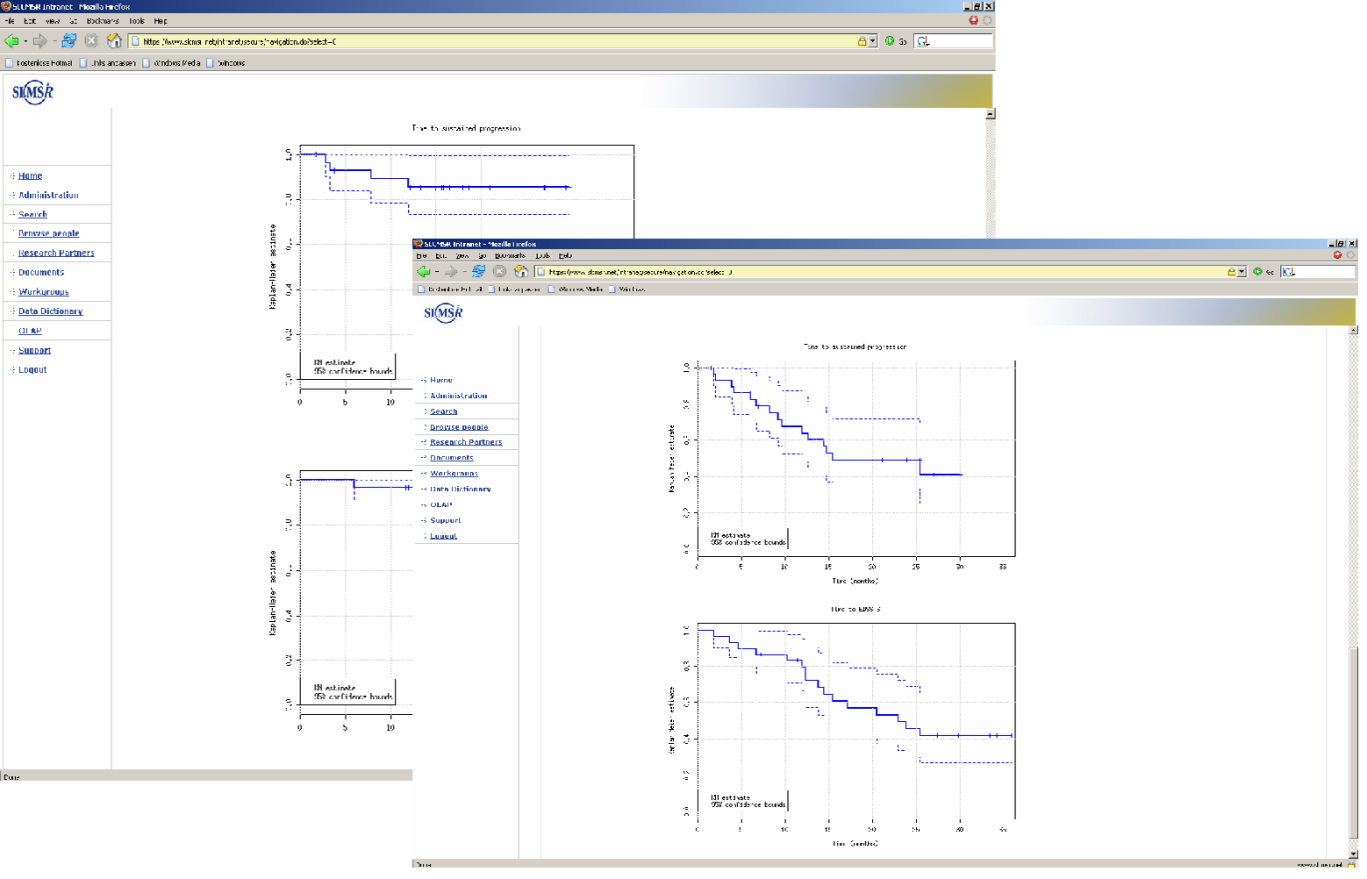
Screenshot from the OLAP-tool "Individual Risk Profile" displaying the Kaplan-Meier curves for the time to sustained progression (6 months confirmation period) and time to EDSS 6. For illustration purposes Kaplan-Meier curves are displayed for a SPMS population. In the background the curves for the population as defined in Figure 1 are displayed.

The patient baseline characteristics of the database are summarized in table 1. The data from patients in the dataset agree well with the literature reports concerning sex ratio, age at disease onset, duration of disease and relapse rate of patients with the same disease course as noted here [6,7]. Demographic information of the selected subgroup is displayed in comparison to the requested characteristics and to demographics of all patients available with the same disease course (see figure 1). Output is provided either as a display of the change of EDSS for individuals in the database over time, regions of progression over time along with the mean relapse rate, or the Kaplan-Meier curve of time to sustained progression in EDSS and time to progression to EDSS 6 (the time of constant assistance to walk) (see figures 2 and 3).

## Discussion

The advantages of OLAP-tools based on a unified database ("data warehousing") are widely applied in business and increasingly used in medical research and medical decision-making. In this report we describe a tool for investigating the course of MS based on specific clinical data and using that information to project disease course in patients of interest.

The predictive power of this tool is limited by restrictions of the composition of patients and duration of observations in the current release of the database. The clinical data are restricted to data from the placebo groups of randomized clinical trials, which may not reflect disease outcomes in patients not involved in clinical trials and certainly do not reflect the impact of daily disease management of patients in non-research clinical settings. Additionally, the observation period of the included patients is limited to a maximum of 3 years (median follow-up 2.2 years) of observation, thus limiting the ability to accurately project longer term outcomes for patients with what is a life-long disease.

A comparison of the tool with population based prevalence cohorts [5-7] is the next step and this will test the reliability of the tool. In the current version the individual datasets consist of the standard variables (course, age at onset, baseline EDSS, disease duration, and number of relapses in the last 12 months). Although available, quantitative MRI variables were not included due to limitations in availability in clinical practise and their relatively poor additional predictive value for near-term clinical outcomes compared to the other clinical variables [12]. Implementing more refined models especially for the development of disease of progressive patients might result in more valid course predictions. Including long-term observational data from natural history studies that cover 20–30 years would help to improve the core data and likely the predictive ability [13,14] of the tool as well, if potentially important predictors for the long-term disease evolution such as time to reach certain EDSS levels (e.g. EDSS = 3, 4 and 6) and time-point of reaching the secondary progressive phase of the disease will be included.

In addition, the utility of the OLAP tool as a predictor of disease course is limited by the patient characteristics in the parent dataset. For instance, there are only a small number of patients with a primary progressive course of MS in the SLCMSR dataset, and this subtype cannot be sufficiently analysed yet. The advantage of the OLAP tool (compared with a purely model based prediction), however, is that this limitation is transparent to the user (figure 1).

## Conclusion

The "individual risk profile" project is based on a comprehensive database and the programming of an OLAP-tool. Our initial intention is to pilot the use of the database amongst neurologists and other researchers experienced in MS with an interest in clinical trials. This phase should provide informed feedback on the utility of the tool for clinical or research use. Updated versions are planned with expanded databases in particular inclusion of long-term natural history data and a broader spectrum of predictors.

Most developmental steps of this project as well as principles of the creation of the underlying database are not disease-specific. It will be of interest to apply the methods and software developed at the SLCMSR for studying MS to other chronic diseases like heart failure, chronic obstructive pulmonary disease, or diabetes mellitus.

## Availability and requirements

**Project name: **OLAP Individual Risk Profile

**Project home page: **

**Operating systems: **Web based application

**Programming language: **R, Java

**Other requirements: **CSS1/UTF-8 compliant browser is required (e.g. Internet Explorer 5.5 or higher, Firefox 1.0 or higher)

**License: **Free, anyone may use the service for non-commercial purposes.

**Any restrictions to use by non-academics: **No.

## Competing interests

The author(s) declare that they have no competing interest.

## Authors' contributions

Martin Daumer and Michael Scholz developed together the idea, Michael Scholz implemented the first version of the tool, and Christian Lederer planned and devised the refined method. Christian Lederer and Anneke Neuhaus programmed the current version of the OLAP-tool. Jerry Wolinsky is chair of the SLC's scientific advisory committee and has tested and evaluated several versions of the tool. All co-authors contributed in critically discussing the overall concept, testing the tool in various stages of its development in on site and during joint conference calls, writing and critical reviewing the manuscript. All coauthors approved the final version of the manuscript.

## Pre-publication history

The pre-publication history for this paper can be accessed here:



## Supplementary Material

Additional File 1 IRPolap.R. The R source code underlying the IRP web application. Click here for file

## Figures and Tables

**Table 1:** Descriptive statistics of clinical trial, placebo arm MS patients derived from the open data set of the SLCMSR and included in "Individual Risk Profile" OLAP-tool, version 2.0.0, divided by the four different courses CIS clinically isolated syndrome, RR relapsing remitting, SP secondary progressive and PP primary progressive.

	**Disease subtype**			
**Database patient characteristics**	**CIS**	**RR**	**SP**	**PP**

**No. of patients**	124	460	455	20
**Sex ratio (m/f)**	0.38	0.41	0.69	0.82
**Age at onset **(Median and 5%- and 95%- percentile)	30 [19;40]	29 [17;41]	29 [18;45]	37 [19;57]
**Baseline EDSS **(Median and 5%- and 95%- percentile)	1.5 [0.0;5.0]	2.5 [1.0;5.5]	5.5 [3.0;6.5]	3.5 [1.5;6.0]
**Duration (months) **(Median and 5%- and 95%- percentile)	2 [0;104]	66 [7;225]	148 [40;341]	50 [11;190]
Relapses last 12 months: **no relapse**	5	47	256	6
Relapses last 12 months: **1 relapse**	115	131	97	4
Relapses last 12 months: **2 or more relapses**	0	132	36	1
Relapses last 12 months: **unknown**	4	150	66	9

The values in brackets are the 5%- and 95%- percentile.
